# Increased Regulatory T Cells and Decreased Myeloid-Derived Suppressor Cells Induced by High CCL17 Levels May Account for Normal Incidence of Cancers among Patients with Atopic Dermatitis

**DOI:** 10.3390/ijms22042025

**Published:** 2021-02-18

**Authors:** Sohshi Morimura, Makoto Sugaya, Tomonori Oka, Hiraku Suga, Tomomitsu Miyagaki, Yuichiro Tsunemi, Yoshihide Asano, Shinichi Sato

**Affiliations:** 1 Department of Dermatology, International University of Health and Welfare, Chiba 286-8520, Japan; sohshimorimura@gmail.com; 2 Department of Dermatology, The University of Tokyo Graduate School of Medicine, Tokyo 113-8655, Japan; tomonorioka16@gmail.com (T.O.); SUGAH-DER@h.u-tokyo.ac.jp (H.S.); asanoyoshihide@hotmail.com (Y.A.); satos-der@h.u-tokyo.ac.jp (S.S.); 3 Department of Dermatology, St. Marianna University School of Medicine, Kanagawa 216-8511, Japan; asahikari1979@gmail.com; 4 Department of Dermatology, Saitama Medical University, Saitama 350-0495, Japan; ytsun-tky@umin.ac.jp

**Keywords:** atopic dermatitis, tumor immunity, CCL17, myeloid-derived suppressor cells

## Abstract

The incidence of cancers in atopic dermatitis (AD) is not increased, although the Th2-dominant environment is known to downregulate tumor immunity. To gain mechanistic insights regarding tumor immunity in AD, we utilized CCL17 transgenic (TG) mice overexpressing CCL17, which is a key chemokine in AD. Tumor formation and lung metastasis were accelerated in CCL17 TG mice when melanoma cells were injected subcutaneously or intravenously. Flow cytometric analysis showed increases in regulatory T cells (Tregs) in lymph nodes in CCL17 TG mice with high mRNA levels of *IL-10* and *Foxp3* in tumors, suggesting that Tregs attenuated tumor immunity. The frequency of myeloid-derived suppressor cells (MDSCs), however, was significantly decreased in tumors of CCL17 TG mice, suggesting that decreased MDSCs might promote tumor immunity. Expression of *CXCL17*, a chemoattractant of MDSCs, was decreased in tumors of CCL17 TG mice. Depletion of Tregs by the anti-CD25 antibody markedly reduced tumor volumes in CCL17 TG mice, suggesting that tumor immunity was accelerated by the decrease in MDSCs in the absence of Tregs. Thus, CCL17 attenuates tumor immunity by increasing Tregs and Th2 cells, while it decreases MDSCs through reductions in CXCL17, which may work as a “safety-net” to reduce the risk of malignant tumors in the Th2-dominant environment.

## 1. Introduction

Atopic dermatitis (AD) is a common skin disorder characterized by an itchy skin rash. The disease is now considered as a complex inflammatory process resulting from activated Th2 cells and disorders of barrier function [1]. Patients with AD have a high risk of allergic comorbidities such as asthma, hay fever, allergic rhinitis, and allergic conjunctivitis [2]. Three clinical patterns (the persistent form, the relapsing form, and the adult-onset form) and various clinical phenotypes have been reported in adult AD [3].

There is increasing epidemiological evidence that AD is generally negatively associated with cancer development. AD has a significantly reduced risk of acute lymphoblastic leukemia and acute myeloid leukemia [4,5]. Most research has shown that there is an inverse association between brain tumors and AD [6,7]. With regard to non-Hodgkin’s lymphoma, one report showed a significantly lower risk in patients with AD [8], while two papers reported a positive association between non-Hodgkin’s lymphoma and AD [9,10]. There was an increased risk of basal cell carcinoma in AD, while no significantly increased risk of malignant melanoma and squamous cell carcinoma [11]. Thus, AD may prevent the development of malignant tumors in some ways, although the Th2-dominant environment is known to downregulate tumor immunity. The underlying mechanism of protecting against malignant tumors in patients with AD is still unclear.

Chemokines are small proteins that stimulate the recruitment of leukocytes. CCL17, known as thymus and activation-regulated chemokine, plays a role in inducing chemotaxis of Th2 cells [12,13]. CCL17 is produced by dendritic cells, T cells, endothelial cells, and fibroblasts. Serum CCL17 levels were found to be significantly higher in patients with AD than in healthy controls [14]. CCL17 is considered as a key chemokine in the development of AD and serves as one of the most reliable biomarkers that can reflect the disease activity of AD. CCL17 has been reported to be related to the development of several other diseases such as cutaneous T-cell lymphoma (CTCL) [15], asthma [16], and acute eosinophilic pneumonia [17]. It has been reported that CCL17 positively regulates tumor development in hepatocellular and gastric carcinomas [18,19]. As CCL17 is overexpressed in AD, it is interesting to focus on the role of CCL17 in tumor immunity.

The tumor microenvironment is an emerging concept that specific types of cells contribute to promoting tumor development. Regulatory T cells (Tregs) have an important function in maintaining the tumor microenvironment [20]. Naïve Tregs develop into effector Tregs after activation by antigens when moving from the thymus to the peripheral blood. Effector Tregs express cytotoxic T-lymphocyte antigen-4 (CTLA-4) to suppress dendritic cell maturation. They also produce cytotoxic substances, such as perforin and granzyme B and cytokines such as TGF-β and IL-10 to suppress cytotoxic T cells, attenuating anti-tumor immunity in the tumor microenvironment. Myeloid derived suppressor cells (MDSCs) are myeloid immature cells that have the ability to suppress T cell responses [21]. MDSCs expand rapidly after activation during infection, cancer, and inflammation. Tumor cells stimulate MDSCs to promote tumor expansion in the tumor microenvironment. We hypothesized that Tregs and/or MDSCs are involved in the development of tumor formation in AD.

Herein, we utilized CCL17 transgenic (TG) mice that were generated in our department [22], to elucidate key functions of CCL17 in tumor immunity in vivo models. The mice overexpressing the CCL17 protein in keratinocytes showed enhanced Th2 responses after antigen challenge, implicating a new AD mouse model. In the present study, to elucidate the role of CCL17 in tumor immunity, skin tumors and lung metastasis were evaluated. As expected, skin tumor formation by melanoma cells and lymphoma cells was significantly enhanced in CCL17 TG mice compared with wild-type (WT) mice. In addition, lung metastasis, made by intravenous injection of melanoma cells, was significantly augmented in CCL17 TG mice. Interestingly, while Tregs were increased in the skin, lymph nodes, and spleen of CCL17 TG mice, MDSCs in the skin tumor were significantly decreased. After Tregs depletion, tumor formation was attenuated in CCL17 TG mice compared with that in WT mice depleted of Tregs, suggesting that the decrease in MDSCs in a high level of CCL17 may positively contribute to tumor immunity in the absence of Tregs. These results implicate that tumor formation may largely depend on the balance of increased Tregs and decreased MDSCs, thereby inducing a normal incidence of tumors in AD.

## 2. Results

### 2.1. Enhanced Skin Tumor Formation in CCL17 TG Mice

**Figure 1:**
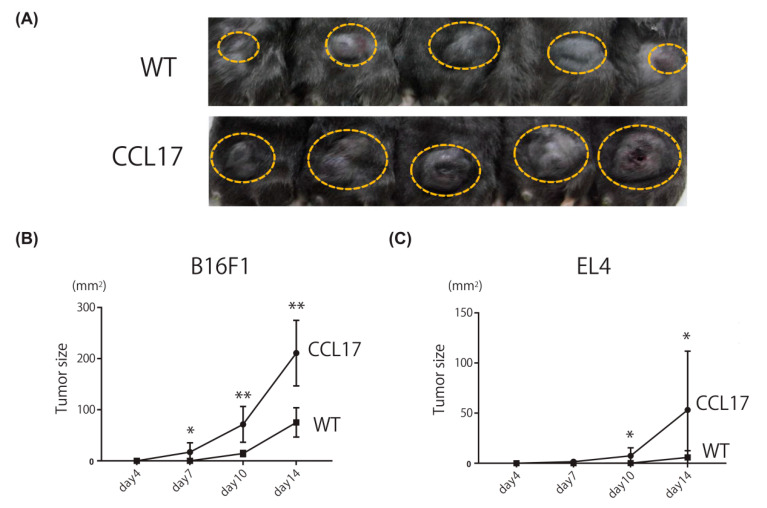
Tumor formation of back skin was enhanced in CCL17 TG mice. Shaved back skin of wild-type (WT) and CCL17 transgenic (TG) mice was treated with injection of B16F1 cells or EL4 cells. The tumor size was calculated using the equation: L1 × L2, where L1 = longest diameter (mm) and L2 = shortest diameter (mm) and evaluated on days 4, 7, 10, and 14. (**A**) Phenotypical manifestation of back skin from WT and CCL17 TG mice 14 days after B16F1 cells injection (yellow circle). (**B**) Tumor sizes were significantly increased in CCL17 TG mice compared with those in WT mice on days 7, 10, and 14 after subcutaneous injection of B16F1 cells. (**C**) Tumor formation was also significantly promoted in CCL17 TG mice compared with that in WT mice on days 10 and 14 after injection of EL4 cells. Data are presented as mean ± SEM of three independent experiments (*n* = 6 for each group). * *p* < 0.05, ** *p* < 0.01, versus WT mice.

We first assessed skin tumor formation of B16 melanoma cells via subcutaneous injection in CCL17 TG mice. Significantly larger tumors were formed in CCL17 TG mice than in WT mice (Figure 1A,B). We next evaluated tumor formation by EL4 mouse lymphoma cells. Tumor formation by EL4 cells in CCL17 TG mice was also enhanced compared with WT mice (Figure 1C). Thus, tumor growth was enhanced where CCL17 was highly expressed.

### 2.2. Promotion of Lung Metastasis in CCL17 TG Mice

**Figure 2:**
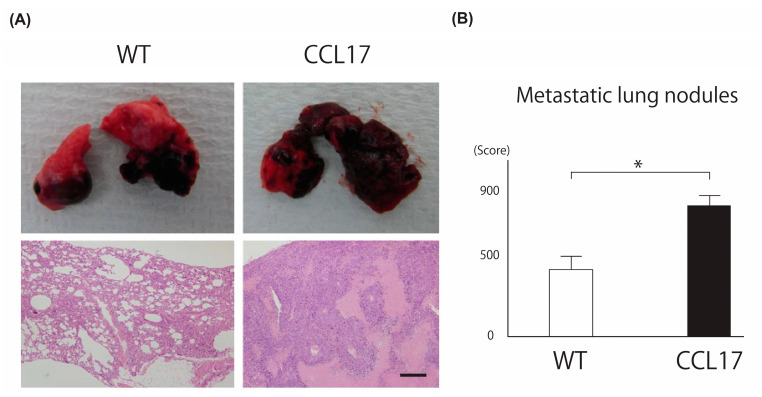
Metastatic lung tumor was promoted in CCL17 TG mice. Wild-type (WT) and CCL17 transgenic (TG) mice were treated with B16F1 melanoma cells injection intravenously. Metastatic lung area was evaluated 21 days after B16F1 cells were injected intravenously. (**A**) Phenotypical manifestation of lungs from WT and CCL17 TG mice 21 days after application of B16F1 cells injection (upper panel). Representative pathological images of lung metastatic clones in WT mice and CCL17 TG mice are shown (lower panel). Original magnification ×100; scale bar = 200 μm. (**B**) Metastatic lung area was significantly larger in CCL17 TG mice compared with that in WT mice. Metastatic lung nodules were evaluated by calculating colony areas as described following. When >60% of the section was occupied with tumor, the colony number was defined as 1000. Each section of the three lobes of the right lung was investigated. Data are presented as mean ± SEM of three independent experiments (*n* = 6 for each group). * *p* < 0.05 WT versus CCL17 TG mice.

We next evaluated lung metastasis by injecting B16 melanoma cells intravenously because the pathophysiology of primary tumor formation and metastasis formation differs. More tumors were formed in CCL17 TG mice than in WT mice after 21 days (Figure 2A upper panel). Histologically, the occupied colony area of tumor cells was significantly larger in CCL17 TG mice than in WT mice (Figure 2A lower panel). We histologically evaluated the number of colonies in each section of the three lobes of the right lung and calculated as the following method that when >60% of the section was occupied with tumor, the colony number was defined as 1000 [23]. Lung metastasis was significantly enhanced at the background of high CCL17.

### 2.3. Increased Tregs in CCL17 TG Mice

**Figure 3:**
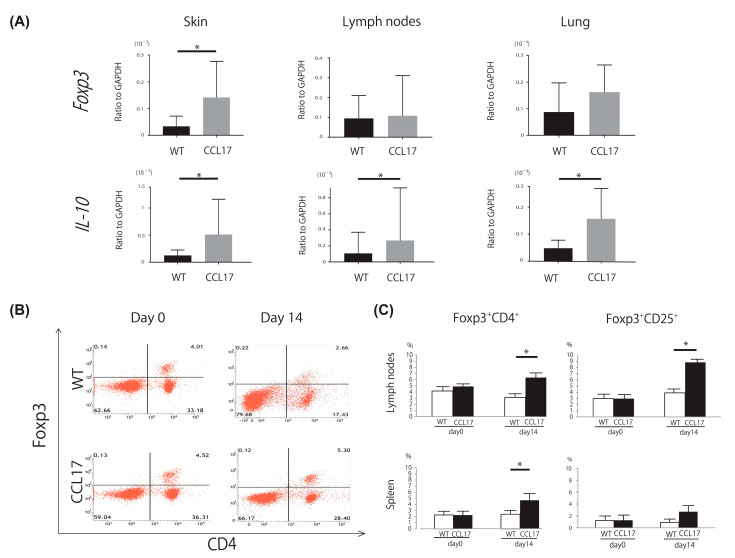
Regulatory T cells (Tregs) were increased in skin tumors, draining lymph nodes, and lung metastasis of CCL17 transgenic (TG) mice. Skin tumors, draining lymph nodes, and spleen were harvested 14 days after subcutaneous injection of B16F1 melanoma cells, while metastatic lung tissues were harvested 21 days after intravenous injection of B16F1 melanoma cells. Expression levels of markers of Tregs were evaluated by quantitative reverse transcription-PCR in skin tumors, draining lymph nodes, and metastatic lung tissues from wild-type (WT) mice and CCL17 TG mice. The frequency of Tregs was analyzed by flowcytometric approach in lymph nodes and spleen from WT mice and CCL17 TG mice. (**A**) *Foxp3* mRNA levels were significantly higher in skin tumors of CCL17 TG mice than those of WT mice. *IL-10* mRNA levels were significantly upregulated in skin tumors, lymph nodes, and metastatic lung tissues of CCL17 TG mice compared with those of WT mice. (**B**) Representative flowcytometric images showing the frequency of Foxp3^+^ CD4^+^ cells in the draining lymph nodes from WT mice and CCL17 mice on days 0 and 14 after tumor injection. (**C**) The frequency of Foxp3^+^ CD4^+^ cells was significantly increased in lymph nodes and spleen on day 14. The frequency of Foxp3^+^ CD25^+^ cells was significantly upregulated in lymph nodes on day 14. Data are presented as mean ± SEM of three independent experiments (*n* = 6 for each group). * *p* < 0.05 WT versus CCL17 TG mice.

Skin tumors and draining lymph nodes were harvested for mRNA expression 14 days after subcutaneous injection of B16 cells. Lung tissues were harvested 21 days after the intravenous injection. Expression levels of *Foxp3* and *IL-10*, both of which are related with Tregs, were evaluated. *Foxp3* mRNA levels were significantly elevated in skin tumors, while *IL-10* mRNA levels were significantly elevated in skin tumors, draining lymph nodes, and lung metastatic nodules in CCL17 TG mice (Figure 3A). Focusing on Tregs, flowcytometric analysis of drain lymph nodes and spleen was performed. The frequencies of Foxp3^+^ CD4^+^ cells and Foxp3^+^ CD25^+^ cells were increased in CCL17 TG mice 14 days after inoculation (Figure 3B,C). Thus, Tregs were increased in draining lymph nodes and the spleen in CCL17 TG mice 14 days after inoculation, which may be responsible for enhanced tumor formation.

### 2.4. Decreased Myeloid-Derived Suppressor Cells in CCL17 TG Mice

**Figure 4:**
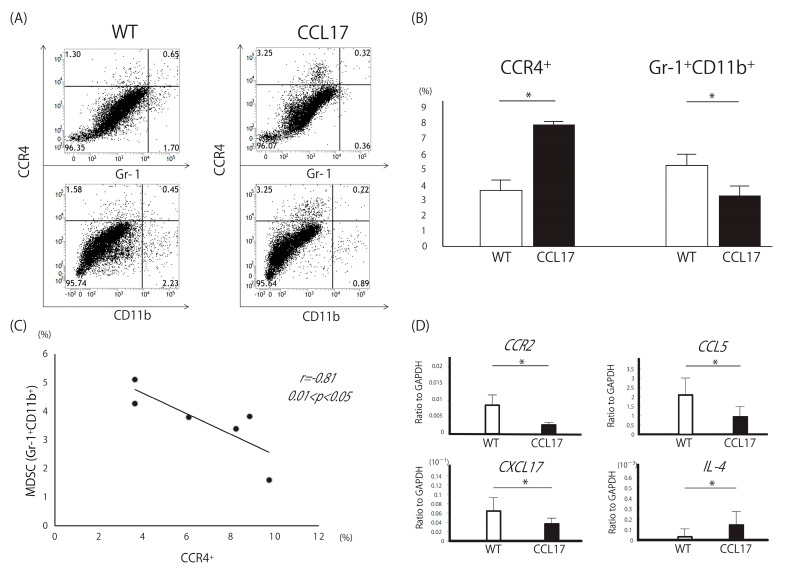
Decreased Myeloid-derived suppressor cells (MDSCs) in CCL17 transgenic (TG) mice. MDSCs are defined as CD11b^+^Gr-1^+^ in mice. CCR4 is expressed by Tregs and Th2 cells. Expression levels of *CCR2*, *CCL5*, and *CXCL17*, which are all related to MDSCs, and *IL-4*, which is produced by Th2 cells, were evaluated by quantitative reverse transcription-PCR in skin tumors from wild-type (WT) and CCL17 TG mice. (**A**) Representative flowcytometric images showing the frequency of CCR4^+^ cells, Gr-1^+^ cells^−^, and CD11b^+^ cells. (**B**) The frequency of CCR4^+^ cells was significantly higher in CCL17 TG mice, while the frequency of CD11b^+^Gr-1^+^ cells was significantly lower compared with that of WT mice, respectively. (**C**) There is a negative correlation between the frequency of CCR4^+^ cells and that of CD11b^+^Gr-1^+^ cells. (**D**) Expression levels of *CCR2*, *CCL5*, and *CXCL17* mRNA were significantly downregulated in CCL17 TG mice, while *IL-4* mRNA levels were significantly upregulated in CCL17 TG mice compared with those in WT mice. * *p* < 0.05 WT versus CCL17 TG mice.

Next, we performed flowcytometric analysis of skin tumors. MDSCs have the capacity to suppress the adaptive immune response mediated by T cells, promoting a tumor-friendly microenvironment. MDSCs are usually defined in mouse models as myeloid cells expressing high levels of CD11b and Gr-1. Therefore, we focused on CCR4^+^, which is expressed by Tregs and Th2 cells, and CD11b^+^Gr-1^+^ population. As expected, the frequency of CCR4^+^ cells was increased in the skin tumors of CCL17 TG mice (Figure 4A,B). On the other hand, the frequency of CD11b^+^Gr-1^+^ cells was significantly decreased in CCL17 TG mice. There was a negative correlation between the frequency of CCR4^+^ cells and that of CD11b^+^Gr-1^+^ cells (Figure 4C).

Messenger RNA expression levels of MDSC-related markers in skin tumors were evaluated. *CXCL17*, *CCR2*, and *CCL5* mRNA levels were significantly decreased in skin tumors of CCL17 TG mice compared with WT mice (Figure 4D). *IL-4* mRNA was increased in CCL17 TG mice, as had been expected. Thus, MDSCs were decreased in the tumor microenvironment in CCL17 TG mice, which might function as a negative regulator preventing severe tumor immune dysfunction.

### 2.5. Enhanced Anti-Tumor Immunity after Depletion of Regulatory T Cells in CCL17 TG Mice

**Figure 5:**
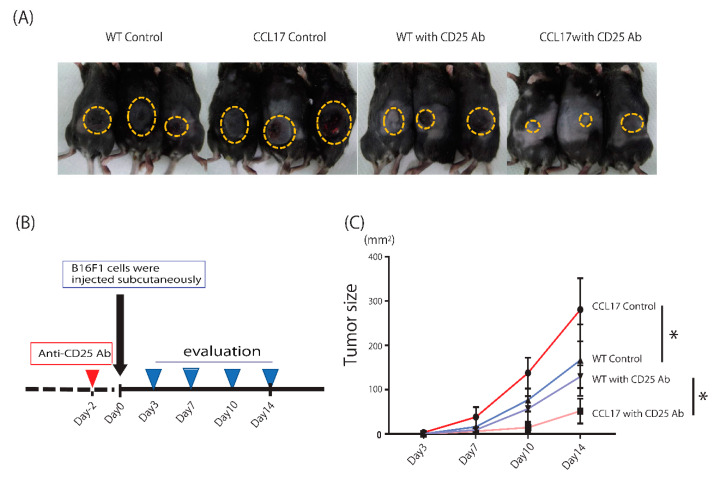
Tumor immunity was markedly enhanced by depletion of regulatory T cells (Tregs) in CCL17 transgenic (TG) mice. (**A**) Phenotypical manifestation of back skin from wild-type (WT) and CCL17 TG mice treated with anti-CD25 Ab or phosphate-buffered saline (PBS) peritoneally 14 days after injection of B16F1 cells subcutaneously. (**B**) Schema describing strategy for Tregs depletion with anti-CD25 Ab. Anti-CD25 Ab was injected peritoneally two days before subcutaneous inoculation of B16F1 cells and tumor sizes were evaluated on days 3, 7, 10, and 14. (**C**) Tumor size was dramatically decreased in CCL17 TG mice treated with anti-CD25 Ab compared with that in WT treated with anti-CD25 Ab and that in CCL17 TG mice treated with PBS. * *p* < 0.05, ** *p* < 0.01.

Tregs promote tumor cell proliferation in the tumor microenvironment. We next determined if depletion of Tregs could change tumor formation in CCL17 TG mice. After peritoneal injection of the anti-CD25 antibody or phosphate-buffered saline (PBS), we evaluated tumor formation 14 days after subcutaneous injection of B16 melanoma cells (Figure 5A,B). As expected, CCL17 TG mice injected with PBS showed larger tumors than WT mice injected with PBS. Depletion of Tregs significantly decreased tumor size both in CCL17 TG mice and WT mice (Figure 5C). Interestingly, tumor formation in CCL17 TG mice was markedly reduced compared with WT mice after depletion of Tregs (Figure 5C). This is probably because decreases in MDSCs in CCL17 TG mice leads to enhanced tumor immunity in the absence of Tregs.

### 2.6. Decrease in CXCL17, a Chemoattractant of MDSCs, in Th2-Dominant Situation

**Figure 6:**
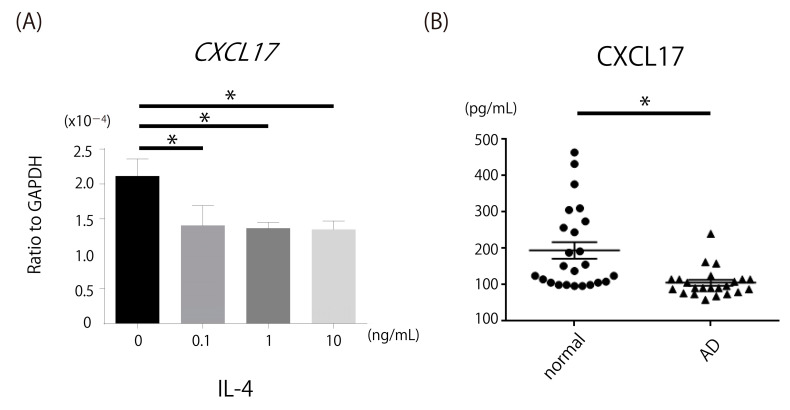
Downregulation of CXCL17 production in IL-4-stimulated normal human epidermal keratinocytes (NHEK) and decreased CXCL17 expression in patients with atopic dermatitis (AD). Expression of *CXCL17* mRNA was assessed in NHEK cells treated with IL-4 stimulation. CXCL17 expression levels were evaluated in sera of patients with AD. (**A**) *CXCL17* mRNA expression by NHEK was significantly attenuated by IL-4 stimulation. (**B**) Serum levels of CXCL17 in patients with AD were significantly decreased compared with normal controls. * *p* < 0.05.

To identify the mechanism by which MDSCs were decreased in CCL17 TG mice, we focused on CXCL17, which induces chemotaxis of MDSCs. IL-4, which was increased in CCL17 TG mice, significantly decreased *CXCL17* mRNA expression by normal human epidermal keratinocytes (NHEK; Figure 6A). Serum levels of CXCL17 in AD patients were significantly decreased compared with normal controls (Figure 6B). Thus, CXCL17 was decreased in Th2-dominant situation, which may account for decreased MDSCs in CCL17 TG mice.

## 3. Discussion

**Figure 7:**
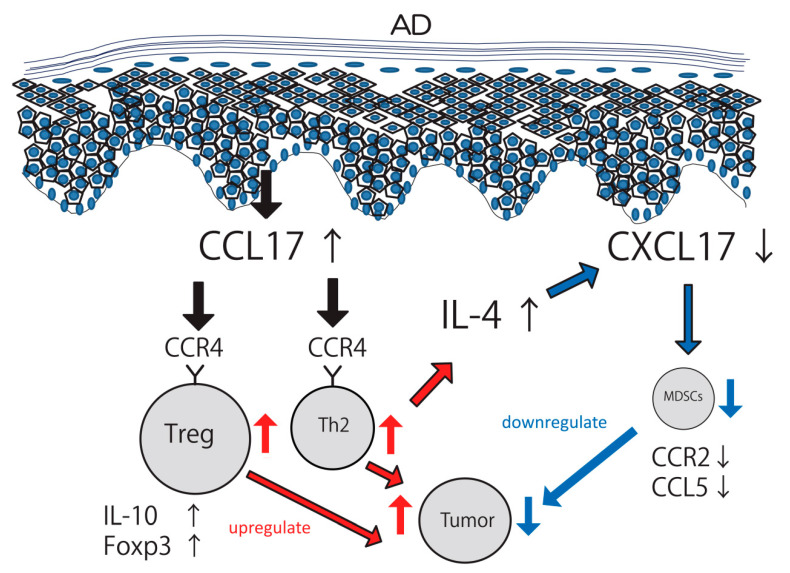
Schema describing relationship among regulatory T cells (Tregs), Th2 cells, and myeloid-derived suppressor cells (MDSCs) in atopic dermatitis (AD). CCL17 is overexpressed in AD and recruits CCR4^+^ cells such as Tregs and Th2 cells into the tumor microenvironment. Tregs, producing IL-10 and Foxp3, attenuate tumor immunity and increase tumor formation. IL-4, which is produced by Th2 cells, downregulates CXCL17 expression. Decreases in CXCL17 lead to reduced frequency of MDSCs, which results in enhanced tumor immunity. Tumor formation is determined by the balance between positive regulation of Tregs and negative regulation of MDSCs.

In the present study, we utilized CCL17 TG mice as a relevant in vivo model for AD to explore the mechanism of tumor immunity. CCL17 TG mice recapitulated key features of AD, demonstrating increased production and secretion of CCL17 [22]. CCL17 overexpression enhanced skin tumor formation and lung metastasis with concomitant increases in Tregs in the skin, lymph nodes, and spleen. The pathophysiology of primary tumor formation and metastasis formation differs. The latter requires multiples steps, such as survival in the blood or lymphatic stream, arrest at distant organ sites, extravasation into the tissues, and proliferation in the foreign microenvironment. Although direct injection of the tumor cell line may not reflect true situations in metastasis, it is still interesting to find that tumor formation is enhanced even in the lung in CCL17 TG mice. The effects of CCL17 should be systemic, not only in the skin, because serum CCL17 levels were extremely high [22]. MDSCs were significantly decreased in CCL17 TG mice and tremendous recovery of tumor immunity was upregulated by deletion of Tregs in CCL17 TG mice, indicating that tumor immunity was accelerated by the decrease in MDSCs in the absence of Tregs. CCL17 has at least two distinct mechanisms to regulate tumor development. In one way, CCL17 increases both Tregs and Th2 cells, upregulating tumor formation. At the same time, CCL17 attenuates MDSCs via a pathway including IL-4 and CXCL17, downregulating tumor formation (Figure 7).

We demonstrated that Th2-type contact hypersensitivity was enhanced and Th1 cells were suppressed in CCL17 TG mice [22]. The frequencies of CCR4^+^ cells and mast cells were high in CCL17 TG mice with increased *IL-4* expression levels. CCL17 modified contact hypersensitivity by attracting CCR4^+^ cells into the skin lesions and generating a Th2-dominant environment. In the present study, the frequency of CCR4^+^ cells in the skin tumor was increased in CCL17 TG mice as had been expected. Interestingly, the frequency of MDSCs was decreased and there was a negative correlation between CCR4^+^ cells and MDSCs. Since CCR4 is expressed by Tregs and Th2 cells, we hypothesized that Tregs and/or Th2 cells could negatively regulate MDSCs directly or indirectly. To gain mechanistic insights, we next focus on CXCL17, a chemoattractant of MDSCs.

CXCL17 is a chemokine known to chemoattract MDSCs, macrophages, and dendritic cells. CXCL17 is strongly expressed by epithelial cells and vascular endothelial cells. There has been increasing evidence that CXCL17 enhances tumor formation in several carcinomas such as lung metastasis [24], hepatocellular carcinoma [25], and colon cancer [26]. Our study showed that *CXCL17* expression was decreased in the skin tumor of CCL17 TG mice, while *IL-4* expression was increased. We next investigated human samples to gain further insights. In sera of AD patients, CXCL17 levels were significantly lower compared with healthy controls. *CXCL17* expression levels were significantly attenuated in NHEK stimulated with IL-4. These results suggest that low levels of CXCL17 results in decreased chemotaxis of MDSCs, eventually leading to promotion of tumor immunity. In Th2-dominant environment, IL-4 production was upregulated by Th2 cells and it may act as a key cytokine to reduce CXCL17 expression, as mentioned previously. In physiological conditions, MDSCs induce chemotaxis of Tregs. In this study, CCL17, a chemoattractant of Tregs, was genetically overexpressed, making it difficult to document direct association between Tregs and MDSCs. Another shortcoming of our study was that we provided only data using primary keratinocytes in vitro. There are other multiple players in the immunological state of the skin, such as dendritic cells, fibroblasts, endothelial cells, resident T cells, and macrophages. Further studies using other cell types would be necessary to elucidate the role of CXCL17 in tumor immunity in the skin.

Several additional publications have suggested an inverse relationship between tumors and AD. The risk of gastric cancer was reduced in patients with AD, especially in males [27]. AD was shown to be associated with a reduced risk of pancreatic cancer in a meta-analysis study [28]. However, none of the articles demonstrated the mechanism by which AD reduced incidence of cancer. Our study indicates that decreases in MDSCs, induced by reductions in CXCL17 as a consequence of CCL17 levels, led to tumor suppression. In the present study, tumor formation was enhanced in CCL17 TG mice. This is probably because the positive impact on tumor formation induced by increased Tregs was much larger than the negative impact by decreased MDSCs in our mouse model. In AD patients, the balance between the positive and negative impacts of tumor formation may be changeable in various circumstances, which may lead to a normal, higher, or lower incidence of malignant tumors, dependent on different types of cancers. IL-4, a representative Th2 cytokine, is also important for maturation of dendritic cells. Mice inoculated with IL-4-producing tumor cells showed rejection and a long-lasting anti-tumor immunity [29]. Therefore, IL-4 itself may have a negative impact on tumor development. Moreover, not only Th2 cytokines, Th17 cytokines and Th1 cytokines are expressed in AD lesional skin [30]. These cytokines may also prevent tumor development.

The treatment of AD has dramatically changed in recent years. Dupilumab, a human monoclonal antibody against IL-4 receptor alpha, blocks signaling of IL-4 and IL-13, improving severe dermatitis in AD patients [31]. This drug is one of the most effective treatments for AD. In the present experiments, we showed that increased production of *IL-4* induced a decrease in *CXCL17*, leading to suppression of tumor formation by blocking the recruitment of MDSCs into the tumor microenvironment. This is probably a protective immune reaction to prevent excessive tumor development. From this aspect, it is important for us to pay attention to the incidence of malignant tumors in AD patients treated with dupilumab. CTCL shares many clinical characteristics with AD, such as a skin rash with severe pruritus and increased serum CCL17, LDH, and IgE levels, making it difficult to distinguish between these diseases [32]. Moreover, some CTCL cases have a history of AD. There have been several papers reporting progression of CTCL after treatment with dupilumab [33,34]. Together with our results, it should be remembered that blocking IL-4/13 signaling may be disadvantageous in some types of malignancy.

In conclusion, we have shown that CCL17 attenuates tumor immunity by increasing the levels of Tregs and Th2 cells, while it decreases MDSCs through CXCL17 reductions. This may be one of the reasons why the incidence of cancers in AD is not increased, working as a “safety-net” to reduce the risk of malignant tumors in the Th2-dominant environment.

## 4. Materials and Methods

### 4.1. Mice

CCL17 TG mice were previously generated in our department [19]. Briefly, CCL17 TG mice show increased production and secretion of the CCL17 protein with biological and functional activity in keratinocytes. All mice were healthy, fertile, and free of signs of infection or disease. Mice used for experiments were 9–12 weeks old. Age-matched wild-type littermates and C57/BL/6 mice were used as controls for CCL17 TG mice. All mice were housed in a pathogen-free barrier facility and they were healthy, fertile, and did not display evidence of infection or disease. All studies and procedures were approved by the Committee an Animal Experimentation of the University of Tokyo.

### 4.2. B16 Melanoma Cells and EL4 Lymphoma Cells

B16 F1 murine melanoma cells and EL4 lymphoma cells were kindly donated by Dr. Sam T Hwang (University of California Davis School of Medicine, Sacramento, CA, USA). They were cultured in MEM medium (Invitrogen, Carsbad, CA, USA) supplemented with 10% heat-inactivated FCS (Harlan)/2 mM (Gemini Bio Products, West Sacramento, CA, USA), L-glutamine /100 units/mL (Wako, Osaka, Japan), penicillin/100 g/mL and streptomycin/0.05 M (Sigma-Aldrich, St Louis, MO, USA), 2-mercaptoethanol (Invitrogen, Carsbad, CA, USA) at 37 °C in 5% CO_2_. Cells were passaged twice a week with trypsin (Sigma-Aldrich, St Louis, MO, USA).

### 4.3. Primary Cutaneous Tumor Growth

B16 F1 cells (5 × 10^6^) in 100 µL of PBS were injected subcutaneously into the shaved lateral flank of anaesthetized mice. The tumor size was calculated using the equation: L1 × L2, where L1 = longest diameter (mm) and L2 = shortest diameter (mm). The size of primary tumors was measured on days 3 or 4, 7, 10, and 14.

### 4.4. Lung Metastasis

B16 F1 cells (5 × 10^6^) in 100 µL of PBS were injected intravenously into the tail vein. The mice were sacrificed on day 21 after injection, and lungs were removed. At the time-point it was not possible to count accurately the number of surface metastatic colonies using a stereomicroscope (Nikon, Tokyo, Japan). because of the small size of the colonies. Therefore, to evaluate lung metastasis, we counted histologically the number of colonies in each section of the three lobes of the right lung. The sections were stained using hematoxylin and eosin (H&E), as described below. When >60% of the section was occupied with tumors, the colony number was defined as 1000 [20]. Each section was examined independently by three investigators in a blinded fashion, and the mean of the results was used for analysis.

### 4.5. RNA Isolation and Quantitative Reverse Transcription-PCR

Skin tumor tissues and lymph nodes were harvested on day 14 after subcutaneous injection of B16F1 melanoma cells. Lung tissues were harvested on day 21 after intravenous injection of B16F1 melanoma cells. RNA was obtained from skin tumor tissues, lymph nodes, and lung tissues with RNeasy Fibrous Tissue Mini Kit (QIAGEN, Valencia, CA, USA). Complementary DNA was synthesized according to the Taqman Gene Expression Protocol (Applied Biosystems, Foster City, CA, USA). Expression mRNA levels of IL-10, Foxp3, CCR2, CCL5, CXCL17, and IL-4 were analyzed using quantitative reverse transcription-PCR method with THUNDERBIRD SYBR qPCR Mix (TOYOBO, Osaka, Japan) on an ABI Prism 7000 sequence detector (Applied Biosystems, Foster City, CA, USA). The mRNA levels were normalized to those of the glyceraldehyde 3-phosphate dehydrogenase (GAPDH) gene. The relative change in the levels of genes of interest was determined by the 2^−ΔΔCT^. Primers used for GAPDH, IL-10, Foxp3, CCR2, CCL5, CXCL17, and IL-4 were as follows: GAPDH forward, 5′-CGT GTT CCT ACC CCC AAT GT-3′ and reverse, 5′-TGT CAT ACT TGG CAG GTT TCT-3′; IL-10 forward, 5′-TTT GAA TTC CCT GGG TGA GAA-3′ and reverse, 5′-ACA GGG GAG AAA TCG ATG ACA -3′; Foxp3 forward, 5′-TAC TTC AAG TTC CAC AAC ATG CGA CC -3′ and reverse, 5′-CGC ACA AAG CAC TTG TGC AGA CTC AG -3′; CCR2 forward, 5′-GTT ACC TCA GTT CAT CCA-3′ and reverse, 5′-CAA GGC TCA CCA TCA TCG TAG TC-3′; CCL5 forward, 5′-CAT ATG GCT CGG ACA CCA-3′ and reverse, 5′-ACA CAC TTG GCG GTT CCT-3′; CXCL17 forward, 5′-TGT TGC TTC CAG TGA TGC TC -3′ and reverse, 5′-GCT GTG GCT TTT CTC TTT GG -3′; human CXCL17 forward, 5′-ACC GAG GCC AGG CTT CTA-3′ and reverse, 5′-GGC TCT CAG GAA CCA ATC TTT-3′, and IL-4 forward, 5′-ACG GAG ATG GAT GTG CCA AAC GTC-3′ and reverse, 5′-CGA GTA ATC CAT TTG CAT GAT GC-3′.

### 4.6. Histologic Examination

Skin tumor tissues and lymph nodes were harvested on day 14 after subcutaneous injection of B16F1 melanoma cells. Lung tissues were harvested on day 21 after intravenous injection of B16F1 melanoma cells. They were formalin (Sigma-Aldrich, St Louis, MO, USA)-fixed and stained with H&E (Sigma-Aldrich, St Louis, MO, USA).

### 4.7. Flow Cytometric Analysis of Lymph Nodes

Draining lymph nodes of the lesional skin (axillary and inguinal lymph nodes) and spleen from WT mice and CCL17 TG mice were harvested on day 14 after subcutaneous injection of B16F1 melanoma cells. Lymph nodes were stained with anti-mouse/rat Foxp3 staining set (71-5775; eBioscience, San Diego, CA, USA), PerCP-Cy TM 5.5-conjugated CD4 (550954; BD Pharmingen, San Diego, CA, USA), PerCP-Cy TM 5.5-conjugated CD25 (551071; BD Pharmingen), PE-conjugated CCR4 (131204; BioLegend, San Diego, CA, USA), FITC-conjugated Gr-1 (108405; BioLegend), and anti-CD11b (101211; BioLegend). Background staining was assessed using non-reactive isotype-matched control monoclonal antibodies (Biolegend). Cells were analyzed on a FACS Verse flow cytometer (BD Biosciences, San Jose, CA, USA).

### 4.8. CXCL17 Expression Analysis of ELISA and In Vitro Cell Culture

Immunoreactive CXCL17 in the serum of patients with AD and healthy controls was quantified by human ELISA kits (MyBioSource, Inc., San Diego, CA, USA). Optical densities were measured at 450 nm, with the correction wavelength set at 570 nm, using a Bio-Rad Model 550 microplate reader (Bio-Rad Laboratories, Hercules, CA, USA). The concentrations were calculated from the standard curve generated by a curve-fitting program according to the manufactures’ instructions.

NHEK were cultured in 75 cm^2^ cell culture flasks (CORNING, Corning, NY, USA) at 37 °C, 5% CO_2_ in Humedia-KB2 (Kurabo Industries, Osaka, Japan) supplemented with Human Keratinocyte Growth Supplement sets Kurabo Industries, Osaka, Japan). When confluence was achieved, the cells were trypsinized, washed, and resuspended in the medium at 5 × 10^5^ cells/mL, and 2 mL was added to each well of the 6-well plates (Becton Dickinson Labware, Franklin Lakes, NJ, USA). When the cells reached semi-confluence, the medium was completely removed and 2 mL of growth supplement-free medium was added to each well. Simultaneously, recombinant human IL-4 (R&D Systems, Minneapolis, MN, USA) was added, and the cells were incubated at 37 °C and 5% CO_2_. The concentration of IL-4 was 0.1, 1, 10 ng/mL. After 24 h, cells were processed by TRIzol Reagent (Invitrogen, Carlsbad, CA, USA) for isolation of total RNA, according to the manufacturers’ instructions.

### 4.9. Statistical Analysis

Data obtained are presented as mean ± SEM. Statistical analysis was carried out with one-way ANOVA with Bonferroni post hoc tests for multiple group comparisons and the two-tailed unpaired t-test for two group comparisons, using Prism Version 7 software [35] (GraphPad, San Diego, CA, USA). For comparing two group values that did not follow Gaussian distribution, the two-tailed Mann–Whitney u test was used. Values of *p* < 0.05 were considered to represent a significant difference.

## Data Availability

The data presented in this study are available on request from the corresponding author. The data are not publicly available due to involvement of clinical samples.

## Abbreviations

AD	atopic dermatitis
CTCL	cutaneous T-cell lymphoma
Tregs	regulatory T cells
TG	transgenic
MDSC	myeloid-derived suppressor cell
WT	wild-type
PBS	phosphate-buffered saline
NHEK	normal human epidermal keratinocytes
GAPDH	glyceraldehyde 3-phosphate dehydrogenase

## References

[1] Weidinger S., Novak N. (2016). Atopic dermatitis. Lancet.

[2] Hanifin J.M. (1984). Atopic dermatitis. J. Allergy Clin. Immunol..

[3] Nettis E., Ortoncelli M., Pellacani G., Foti C., Di Leo E., Patruno C., Rongioletti F., Argenziano G., Ferrucci S.M., Macchia L. (2020). A Multicenter Study on the Prevalence of Clinical Patterns and Clinical Phenotypes in Adult Atopic Dermatitis. J. Investig. Allergol. Clin. Immunol..

[4] Schüz J., Morgan G., Böhler E., Kaatsch P., Michaelis J., Münster E. (2003). Atopic disease and childhood acute lymphoblastic leukemia. Int. J. Cancer.

[5] Wen W., Shu X.O., Linet M.S., Neglia J.P., Potter J.D., Trigg M.E., Robison L.L. (2000). Allergic disorders and the risk of childhood acute lymphoblastic leukemia (United States). Cancer Causes Control..

[6] Schlehofer B., Blettner M., Preston-Martin S., Niehoff D., Arslan A., Ahlbom A., Choi W.N., Giles G.G., Howe G.R., Little J. (1999). Role of medical history in brain tumour development. Results from the international adult brain tumour study. Int. J. Cancer.

[7] Brenner A.V., Linet M.S., Fine H.A., Shapiro W.R., Selker R.G., Black P.M., Inskip P.D. (2002). History of allergies and autoimmune diseases and risk of brain tumors in adults. Int. J. Cancer.

[8] Fabbro-Peray P., Daures J.-P., Rossi J.-F. (2001). Environmental risk factors for non-Hodgkin’s lymphoma: A population-based case-control study in Languedoc-Roussillon, France. Cancer Causes Control..

[9] Doody M.M., Linet M.S., Glass A.G., Friedman G.D., Pottern L.M., Boice J.D., Fraumeni J.F. (1992). Leukemia, lymphoma, and multiple myeloma following selected medical conditions. Cancer Causes Control..

[10] Cartwright R., McKinney P., O’Brien C., Richards I., Roberts B., Lauder I., Darwin C., Bernard S., Bird C. (1988). Non-hodgkin’s lymphoma: Case control epidemiological study in Yorkshire. Leuk. Res..

[11] Gandini S., Stanganell I., Palli D., Giorgi V.D., Masala G., Caini S. (2016). Atopic dermatitis, naevi count and skin cancer risk: A meta-analysis. J. Dermatol. Sci..

[12] Imai T., Baba M., Nishimura M., Kakizaki M., Takagi S., Yoshie O. (1997). The T cell-directed CC chemokine TARC is a higly specific biological ligand for C chemokine receptor 4. J. Biol. Chem..

[13] Zlotnik A., Yoshie O. (2000). Chemokines: A new classification system and their role in immunity. Immunity.

[14] Kakinuma T., Nakamura K., Wakugawa M., Mitsui H., Tada Y., Saeki H., Torii H., Asahina A., Onai N., Matsushima K. (2001). Thymus and activation-regulated chemokine in atopic dermatitis: Serum thymus and activation-regulated chemokine level is closely related with disease activity. J. Allergy Clin. Immunol..

[15] Kakinuma T., Sugaya M., Nakamura K., Kaneko F., Wakugawa M., Matsushima K., Tamaki K. (2003). Thymus and activa-tion-regulated chemokine (TARC/CCL17) in mycosis fungoides: Serum TARC levels reflect the disease activity of mycosis fungoides. J. Am. Acad. Dermatol..

[16] Sekiya T., Yamada H., Yamaguchi M., Yamamoto K., Ishii A., Yoshie O., Sano Y., Morita A., Matsushima K., Hirai K. (2002). Increased levels of a TH2-type CC chemokine thymus and activation-regulated chemokine (TARC) in serum and induced sputum of asthmatics. Allergy.

[17] Miyazaki E., Nureki S., Fukami T., Shigenaga T., Ando M., Ito K., Ando H., Sugisaki K., Kumamoto T., Tsuda T. (2002). Elevated levels of thymus-and actication-regulated chemokine in brochoalveolar lavage fluid from patients with eosinophilic pneumonia. Am. J. Respir. Crit. Care Med..

[18] Zhu F., Li X., Chen S., Zeng Q., Zhao Y., Luo F. (2016). Tumor-associated macrophage or chemokine ligand CCL17 positively regulates the tumorigenesis of hepatocellular carcinoma. Med. Oncol..

[19] Mizukami Y., Kono K., Kawaguchi Y., Akaike H., Kamimura K., Sugai H., Fujii H. (2008). CCL17 and CCL22 chemokines within tumor microenvironment are related to accumulation of Foxp3+ regulatory T cells in gastric cancer. Int. J. Cancer.

[20] Strauss L., Bergmann C., Szczepanski M., Gooding W., Johnson J.T., Whiteside T.L. (2007). A unique subset of CD4+CD25highFoxp3+ T cells secreting interleukin-10 and transforming growth factor-beta1 mediates suppression in the tumor microenvironment. Clin. Cancer Res..

[21] Ostrand-Rosenberg S., Sinha P. (2009). Myeloid-Derived Suppressor Cells: Linking Inflammation and Cancer. J. Immunol..

[22] Tsunemi Y., Saeki H., Nakamura K., Nagakubo D., Nakayama T., Yoshie O., Kagami S., Shimazu K., Kadono T., Sugaya M. (2006). CCL17 transgenic mice show an enhanced Th2-type response to both allergic and non-allergic stimuli. Eur. J. Immunol..

[23] Yamada M., Yanaba K., Hasegawa M., Matsushita Y., Horikawa M., Komura K., Matsushita T., Kawasuji A., Fujita T., Takehara K. (2005). Regulation of local and metastatic host-mediated anti-tumour mechanisms by l-selectin and intercellular adhesion molecule-1. Clin. Exp. Immunol..

[24] Hsu Y.-L., Yen M.-C., Chang W.-A., Tsai P.-H., Pan Y.-C., Liao S.-H., Kuo P.-L. (2019). CXCL17-derived CD11b+Gr-1+ myeloid-derived suppressor cells contribute to lung metastasis of breast cancer through platelet-derived growth factor-BB. Breast Cancer Res..

[25] Li L., Yan J., Xu J., Liu C.-Q., Zhen Z.-J., Chen H.-W., Ji Y., Wu Z.-P., Hu J.-Y., Zheng L. (2014). CXCL17 Expression Predicts Poor Prognosis and Correlates with Adverse Immune Infiltration in Hepatocellular Carcinoma. PLoS ONE.

[26] Ohlsson L., Hammarström M.-L., Lindmark G., Hammarström S., Sitohy B. (2016). Ectopic expression of the chemokine CXCL17 in colon cancer cells. Br. J. Cancer.

[27] Jo S., Kim T.J., Lee H., Min Y.W., Min B.H., Lee J.H., Son H.J., Rhee P.L., Beak S.Y., Kim S.W. (2018). Association be-tween Atopic Dermatitis and Risk of Gastric Cancer: A Nationwide Population-based Study. Korean J. Gastroenterol..

[28] Gandini S., Lowenfels A.B., Jaffee E.M., Armstrong T.D., Maisonneuve P. (2005). Allergies and the Risk of Pancreatic Cancer: A Meta-analysis with Review of Epidemiology and Biological Mechanisms. Cancer Epidemiol. Biomark. Prev..

[29] Golumbek P.T., Lazenby A.J., I Levitsky H., Jaffee L.M., Karasuyama H., Baker M., Pardoll D.M. (1991). Treatment of established renal cancer by tumor cells engineered to secrete interleukin-4. Science.

[30] Noda S., Suárez-Fariñas M., Ungar B., Kim S.J., Strong C.D.G., Xu H., Peng X., Estrada Y.D., Nakajima S., Honda T. (2015). The Asian atopic dermatitis phenotype combines features of atopic dermatitis and psoriasis with increased TH17 polarization. J. Allergy Clin. Immunol..

[31] Gooderham M.J., Hong H.C.-H., Eshtiaghi P., Papp K.A. (2018). Dupilumab: A review of its use in the treatment of atopic dermatitis. J. Am. Acad. Dermatol..

[32] Miyagaki T., Sugaya M. (2011). Erythrodermic cutaneous T-cell lymphoma: How to differentiate this rare disease from atopic der-matitis. J. Dermatol. Sci..

[33] Espinosa M.L., Nguyen M.T., Aguirre A.S., Martinez-Escala M.E., Kim J., Walker C.J., Pontes D.S., Silverberg J.I., Choi J., Pro B. (2020). Progression of cutaneous T-cell lymphoma after dupilumab: Case review of 7 patients. J. Am. Acad. Dermatol..

[34] Hollins L.C., Wirth P., Fulchiero G.J., Foulke G.T. (2020). Long-standing dermatitis treated with dupilumab with subsequent pro-gression to cutaneous T-cell lymphoma. Cutis.

[35] Harvey M. (1994). Prism, Version 7.

